# Moderate thinning enhances soil water and its temporal stability in Chinese pine plantations on the semi-arid Loess Plateau of China

**DOI:** 10.3389/fpls.2026.1805676

**Published:** 2026-04-29

**Authors:** Qindi Zhang, Yanqing Zhang, Jiaying Cao, Zongshan Li, Shengnan Chen, Wei Wei, Zhenfeng Li

**Affiliations:** 1 Research Center for Ecological Restoration, School of Life Sciences, Shanxi Normal University, Taiyuan, China; 2 State Key Laboratory of Regional and Urban Ecology, Research Center for Eco-Environmental Sciences, Chinese Academy of Sciences, Beijing, China; 3 National Observation and Research Station of Earth Critical Zone on the Loess Plateau in Shaanxi, Xi’an, China; 4 Chankou Forestry Experimental Station of Dingxi City, Dingxi, China

**Keywords:** Chinese pine, coefficient of variation, loess plateau, soil water content, thinning

## Abstract

**Introduction:**

Water scarcity severely constrains the sustainability of plantations in semi-arid regions by reducing soil water availability and increasing drought stress. Thinning is a crucial silvicultural practice for forest restoration and water regulation, yet the responses of soil water content (SWC) to varying thinning intensities during a dry year and a normal year remain insufficiently understood.

**Methods:**

This study evaluated the effects of thinning on SWC in Chinese pine (*Pinus tabuliformis*) plantations on the Loess Plateau of China, with five thinning intensities: 0% (control), 15% (light), 30% (moderate), 45% (heavy), and 60% (extremely heavy). SWC at depths of 0 -200 cm was repeatedly monitored during the growing seasons of 2023 (dry year) and 2024 (normal year). Vegetation structure, rainfall redistribution, and soil properties were periodically measured.

**Results:**

Thinning increased mean 0 -200 cm profile SWC by 4.96 -18.06% in the dry year (2023) and by 5.33 -18.87% in the normal year (2024), compared with the 0% thinning control. Although SWC in 2024 was higher than in 2023, thinning exerted a similar effect on SWC in both study years, with the 30% thinning intensity yielding the most pronounced positive improvement in deep SWC (30 -200 cm; +19.14% -25.26%). Thinning significantly improved the temporal stability of deep SWC in 2024, and the 15% and 30% thinning treatments consistently exhibited relatively low coefficient of variation values during the monitored observation period. Redundancy analysis (RDA) indicated that SWC was positively associated with net precipitation in both soil layers and in both study years. Additionally, deep SWC exhibited a positive association with soil organic carbon during both years, as well as with fine-root traits in 2024.

**Discussion:**

Based on the above results and the regional soil water carrying capacity, we recommend prioritizing an initial thinning intensity of approximately 30% (≈2,000 stems ha−1) to alleviate soil desiccation in Chinese pine plantations. This finding informs adaptive eco-rehabilitation strategies for plantation restoration on the Loess Plateau and other semi-arid regions and provides a practical basis for improving soil-water regulation and plantation resilience under increasing climatic stress.

## 1 Introduction

Soil water is a key variable for regulating ecohydrological processes and the soil–plant–atmosphere continuum, and it is critical for maintaining the functioning of terrestrial ecosystems (Vereecken et al., 2015; Legates et al., 2010). In semi-arid regions, establishing large-scale, high-density monoculture plantations can effectively promote land greening and enhance specific ecosystem services, often at the cost of soil water decline (Jasechko et al., 2013; Filoso et al., 2017). Moreover, increased water consumption and intensified resource competition tend to further exacerbate regional water deficits and weaken the stability of ecosystem restoration over time (Liu et al., 2018; Tang et al., 2016; Farley et al., 2005). To mitigate this risk, optimizing community structure and reducing stand density to alleviate water deficit constitute a key strategy for safeguarding soil water security and enhancing plantation sustainability.

As a key eco-rehabilitation measure in water-limited regions, thinning can regulate soil water mainly by reducing stand density and increasing net precipitation inputs, thereby enhancing infiltration and soil water recharge (Xiao et al., 2025; Ding et al., 2025). Accordingly, the effects of thinning on SWC are likely to differ across thinning intensities, suggesting that an optimal thinning intensity may exist (Wang et al., 2021). For example, a global synthesis suggests that an approximately 50% reduction in stand density represents a common threshold for pronounced hydrological responses (del Campo et al., 2022). Meanwhile, the effects of thinning on soil water often differ considerably across different soil layers within the profile (Zhu et al., 2017; Liu et al., 2024). This layer-specific discrepancy can be attributed to the rapid response of the surface layer to rainfall inputs, whereas the deep layer typically exhibits a lagged response due to delayed percolation recharge (Zhang et al., 2024; da Silva-Dias et al., 2024). Furthermore, hydrological conditions modulate the spatiotemporal dynamics of SWC by altering the soil water budget. In normal years, frequent rainfall inputs sustain infiltration and percolation recharge, thus maintaining relatively high and temporally stable SWC (Liu and Shao, 2015). In contrast, SWC is characterized by low levels and high temporal variability in dry years (Yuan et al., 2026). Plantations with stable soil moisture regimes exhibit markedly enhanced drought resistance, which bolsters the resilience of plantation ecosystems and effectively mitigates stand damage induced by drought stress (Collado et al., 2023). Given the frequent occurrence of drought, it remains unclear whether optimized thinning thresholds should be identified primarily from SWC responses under dry conditions rather than under normal hydrological conditions. However, the dynamic responses of profile SWC to thinning, and the associated optimized thinning thresholds, have not been sufficiently quantified under contrasting hydrological conditions represented by a dry year and a normal year.

The Loess Plateau is a typical semi-arid region of China, where large areas of Chinese pine (*Pinus tabuliformis*) plantations have been established for ecological restoration (Liu et al., 2024; Cheng et al., 2023). However, many plantation initiatives have adopted monoculture practices, which in turn induce soil water deficits, hinder tree growth, and degrade ecosystem functions (Li et al., 2023; Bremer and Farley, 2010). Although thinning is widely recognized as a core eco-rehabilitation measure, optimized thinning thresholds for mitigating soil desiccation remain inadequately quantified. To address these issues, we established a gradient thinning experiment in a typical Chinese pine plantation on the semi-arid Loess Plateau and monitored SWC dynamics in a dry year (2023) and a normal year (2024). We hypothesized that SWC responses to thinning would differ between the dry year and the normal year, leading to different optimized thinning thresholds. The objectives were to: (1) characterize SWC responses to varying thinning intensities in a dry year and a normal year; (2) identify the optimized thinning thresholds and residual stand densities for soil water security. These findings will provide critical scientific support for sustainable management of plantations and ecosystem restoration on the Loess Plateau and other water-limited regions.

## 2 Materials and methods

### 2.1 Study area

**Figure 1:**
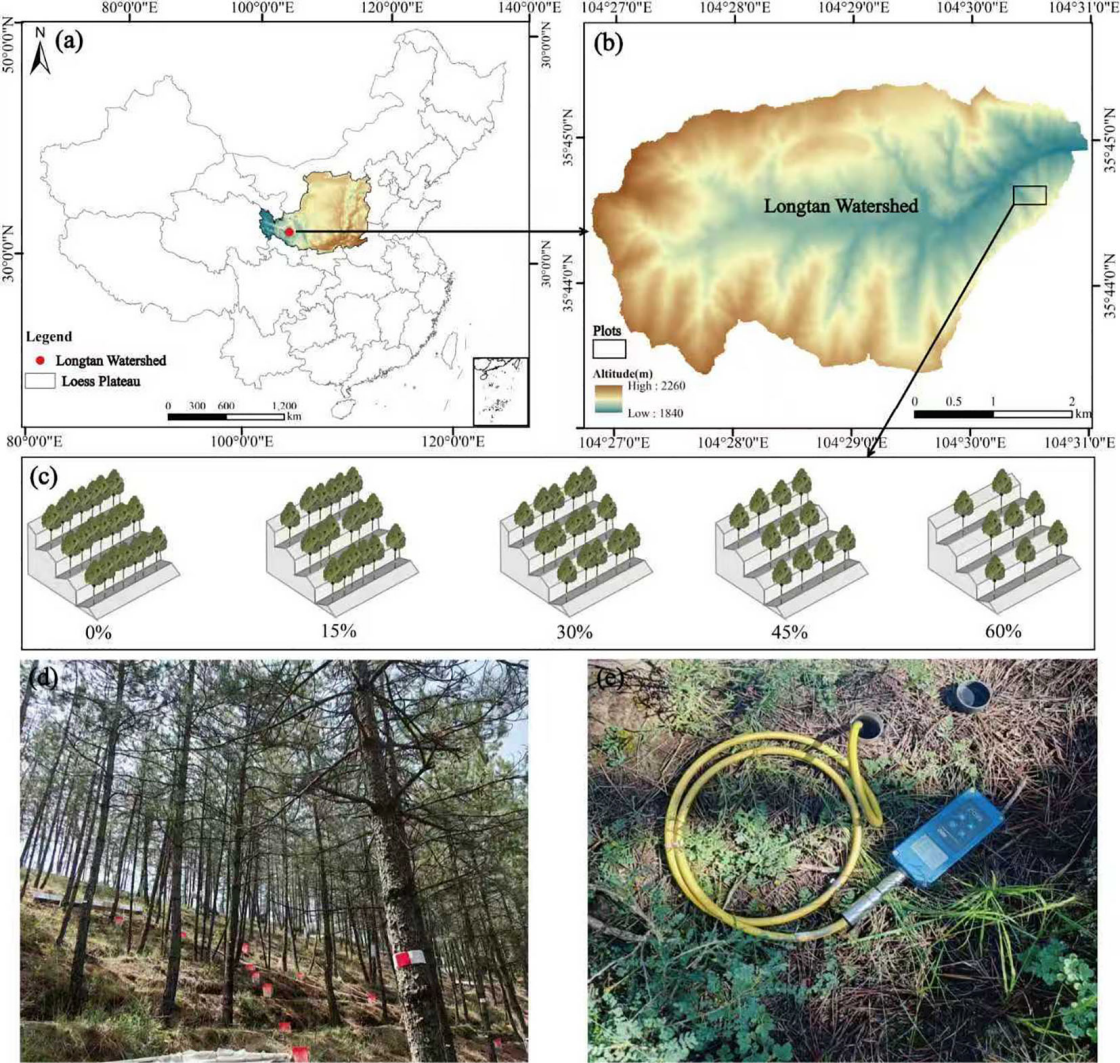
Location of the study area and experimental design. **(A)** Location of the Longtan watershed within China and the Loess Plateau. **(B)** Topography of the Longtan watershed and location of the experimental plots. **(C)** Schematic diagram of the five thinning treatments: 0% (control), 15% (light), 30% (moderate), 45% (heavy), and 60% (extremely heavy), corresponding to residual stand densities of 2844, 2404, 2000, 1556, and 1124 stems ha^−1^, respectively. **(D)** Photograph of the experimental plots. **(E)** Portable TDR used for SWC monitoring.

The study area is located in the Longtan catchment (35°43′–35°46′N, 104°27′–104°31′E), Dingxi City, Gansu Province, China (**Figures 1A, B**), which represents a typical loess hilly region of the Loess Plateau. It is characterized by fragmented terrain and undulating hills, constituting a semi-arid continental climate zone. The mean annual temperature is 6.8 °C, with average precipitation of 386 mm concentrated predominantly between June and September. The elevations range from 1834 to 2233 m, with loess soil as the dominant type. Soil texture comprises approximately 50% silt, 39% sand, and 11% clay. The soil water holding capacity ranges from 0.18 to 0.24 g/g, with a permanent wilting point of 0.05 g/g (Wei et al., 2019). Artificial vegetation dominates the area. The main plantings are Chinese pine, Chinese arborvitae (*Platycladus orientalis*), Siberian apricot (*Armeniaca sibirica*), Korshinsk peashrub (*Caragana korshinskii*), and alfalfa (*Medicago sativa*). The Chinese pine plantations, established in 1972 at an initial density of 2844 stems ha^-1^, have subsequently developed homogeneous species composition, tree growth decline, and soil desiccation (Liu et al., 2025).

### 2.2 Experimental design

In 2019, we established five parallel sample plots (30 m × 30 m) in Chinese pine plantations. All plots were located on sites with identical slope aspect and gradient and prepared with counter-slope terraces (**Figures 1C, D**). Thinning treatments were applied at five intensities: 0% (control), 15% (light), 30% (moderate), 45% (heavy), and 60% (extremely heavy). The residual densities were 2844, 2404, 2000, 1556, and 1124 stems ha^-1^, respectively.

### 2.3 Soil water measurements

SWC within each plot was monitored using a portable time-domain reflectometer (TDR; TRIME-FM, IMKO, Ettlingen, Germany; **Figure 1E**). Each plot contained three permanently installed access cylindrical tubes. SWC was measured at 20 cm intervals from 0 to 200 cm. Measurements were conducted on multiple observation dates at approximately semi-monthly intervals, with available observations spanning mid-April to mid-October in 2023 and early July to early October in 2024. The coefficient of variation (CV, %) of SWC was calculated across repeated observation dates within each year to quantify relative variability and temporal stability. A lower CV indicates greater temporal stability. Data from May to June 2024 were missing due to instrument malfunctions. To preserve the authenticity and comparability of the observed data, no interpolation or imputation was applied to these missing observations. For 2024, layer-specific mean SWC values and corresponding CV estimates were calculated using only the available observations after instrument recovery. Thus, comparisons between 2023 and 2024 should be interpreted as comparisons within the monitored observation period rather than as full growing-season contrasts.

### 2.4 Environmental factor measurements

In each thinning plot, we measured aboveground and belowground environmental factors related to SWC, including net precipitation, fine-root traits, understory plant diversity, and soil organic carbon content. These variables were measured during the growing seasons (May–September) of 2023 and 2024.

Net precipitation (NP, mm) was defined as the sum of throughfall and stemflow. Throughfall (mm) was recorded using 21 rain gauges evenly distributed along the diagonal of each plot, and stemflow (mm) was measured with collar collectors installed on four tree trunks. Net precipitation was calculated as the average throughfall plus stemflow across 40 rainfall events over the two-year period.

Fine root traits were measured using the soil profile method. Soil samples were collected at 10 cm intervals to a depth of 100 cm using a 100 cm³ core sampler. After cleaning, root systems were scanned with the WinRHIZO root analysis system (version 2009b, Regent Instruments Inc., Canada). Root traits, including specific surface area (SSA, cm² g^-^¹), specific root length (SRL, m g^-^¹), fine root biomass (FRB, g m^-^³), fine root average diameter (FRAD, mm), and root surface area density (RSAD, m² m^-^³), were quantified (Jia et al., 2021; Zhang et al., 2015).

Understory plant diversity was assessed through field surveys employing the quadrat method. Within each plot, six 1 m × 1 m subplots were established along the diagonal. For each subplot, plant species composition and percent cover were recorded. The Patrick richness index (*S*), Shannon–Wiener diversity index (*H*′), Simpson dominance index (*D*), and Pielou evenness index (*J*) were calculated as follows:


R=S

$$H^{\prime }=-\sum_{i=1}^{S}P_{i}\cdot \ln P_{i}$$

$$D=1-\sum_{i=1}^{S}P_{i}^{2}\;$$

$$J=H^{\prime }/\ln S$$

where *S* is the total number of species in the quadrat, and *P_i_* is the importance value.

Soil samples at the 0–100 cm depths were collected using a soil auger (5 cm diameter) at 20 cm intervals. For each plot, three soil cores were sampled at each soil depth along a diagonal line, and then thoroughly mixed to form one composite sample. Soil organic carbon (SOC, %) was then determined by the potassium dichromate oxidation method with external heating.

### 2.5 Empirical frequency analysis to divide hydrological years

**Figure 2:**
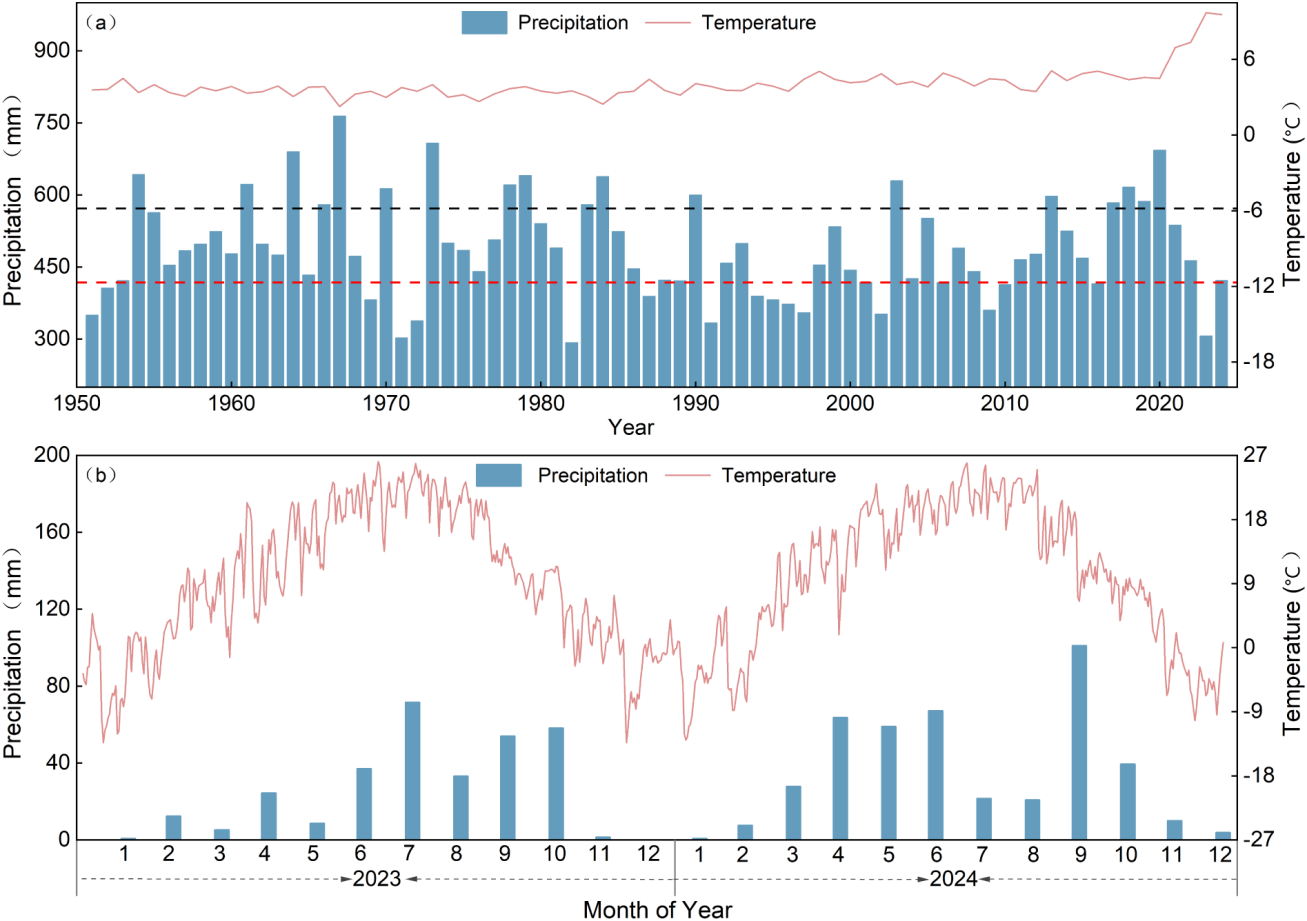
Analysis of precipitation and temperature change trends. **(A)** Annual precipitation and mean annual temperature from 1950 to 2024. The red dashed line indicates the dry-year threshold derived from empirical frequency analysis (P = 75%, 418.3 mm), whereas the black dashed line indicates the wet-year threshold (P = 25%, 567.7 mm). Years with annual precipitation below the red dashed line were classified as dry years, those above the black dashed line were classified as wet years, and those in between were classified as normal years. **(B)** Monthly precipitation and air temperature in 2023 and 2024.

Hydrological years were delineated based on annual precipitation from 1950 to 2024 (**Figures 2A, B**). The annual precipitation totals in 2023 and 2024 were 255.6 mm and 422.6 mm, respectively. Following the methodology of Zhao et al. (2020), 2023 was designated the dry year and 2024 the normal year.

### 2.6 Soil layer partitioning and profile-scale SWC calculation

To characterize both depth-specific responses and the integrated whole-profile response of soil water content, SWC was summarized separately for the surface layer (0–30 cm) and the deep layer (30–200 cm), and the mean SWC of the entire 0–200 cm profile was also calculated. The 0–30 cm interval was defined as the surface layer because previous studies in water-limited ecosystems, as well as the temporal patterns observed in this study, indicate that this layer is more directly influenced by precipitation input and surface evaporation. The 30–200 cm interval was defined as the deep layer because it is less directly affected by short-term atmospheric forcing and better represents subsurface water storage dynamics within the soil profile. In addition, the 0–200 cm mean SWC was retained as an integrated indicator of the net whole-profile response to thinning, rather than being interpreted as an independent functional soil layer. This depth-based framework was used to distinguish near-surface moisture dynamics directly influenced by precipitation input from deeper profile-scale water storage responses, while also providing an overall measure of treatment effects across the monitored profile (Jian et al., 2015; Wang et al., 2024).

### 2.7 Statistical analyses

To characterize the SWC profile, SWC and its vertical variation were calculated at 20-cm intervals, and the mean values were used for subsequent analyses. Before repeated-measures analysis of variance (ANOVA), normality and homogeneity of variance were tested using the Shapiro–Wilk test and Levene’s test. The significance level was set at (*P <* 0.05). Bar charts depict means ± standard error (SE). Additionally, redundancy analysis (RDA) was employed to evaluate the effects of thinning intensities on SWC at each layer via their influence on key regulatory environmental factors. For *post hoc* comparisons, when the interaction was significant, simple-effects tests with multiple-comparison adjustment (e.g., Bonferroni) were applied; when the interaction was non-significant, Tukey’s HSD was used for main-effect means. All statistical analyses and plotting were conducted in R version 4.5.0.

## 3 Results

### 3.1 Vertical variation and layer-specific SWC responses to thinning intensity

**Figure 3:**
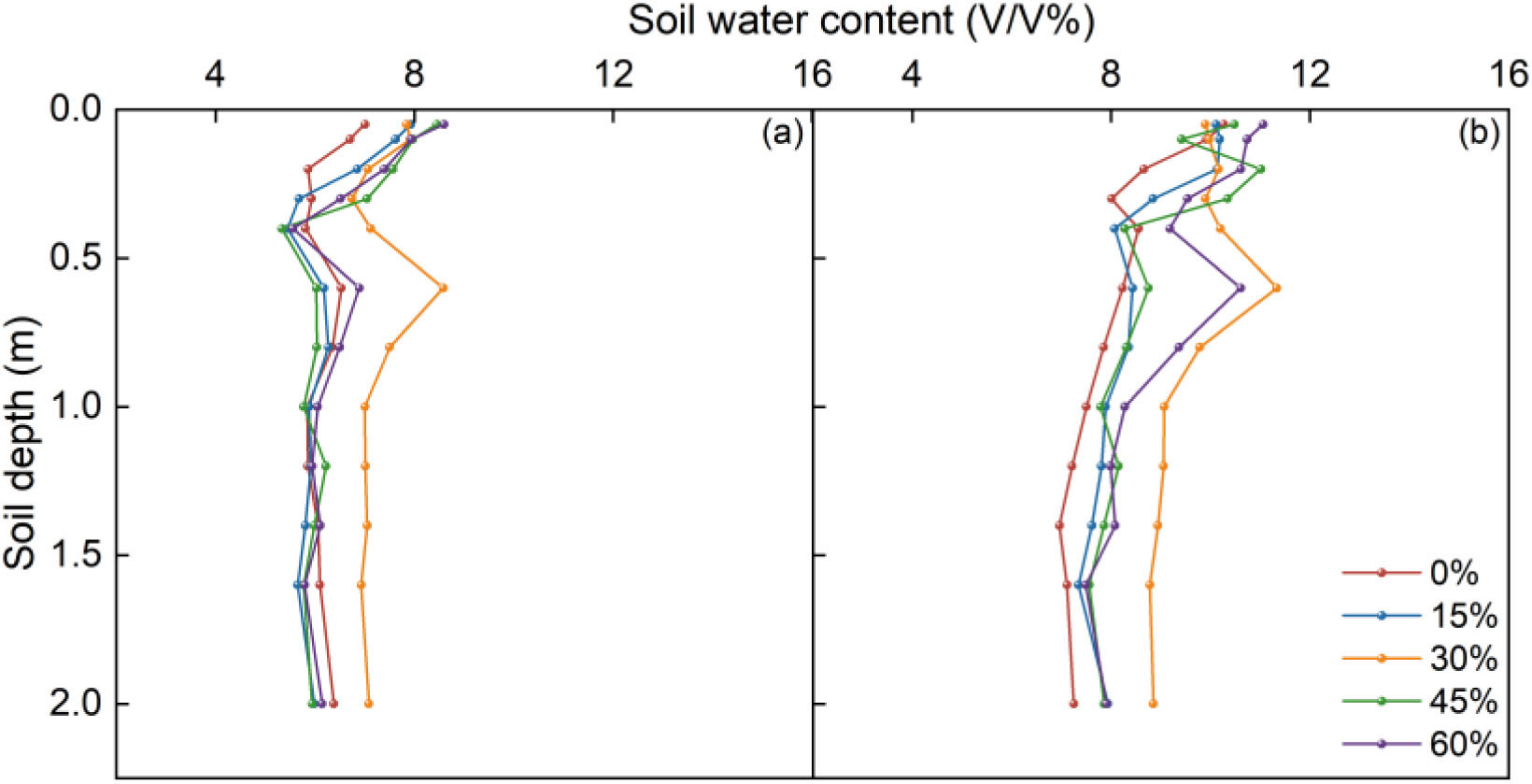
Vertical profiles of mean SWC at different thinning intensities during the monitored observation period in 2023 **(A)** and 2024 **(B)**. The percentages (0%, 15%, 30%, 45%, and 60%) denote thinning intensity, i.e., the proportion of stems removed relative to the pre-thinning stand density. These treatments correspond to residual stand densities of 2844, 2404, 2000, 1556, and 1124 stems ha^−1^, respectively.

**Figure 4:**
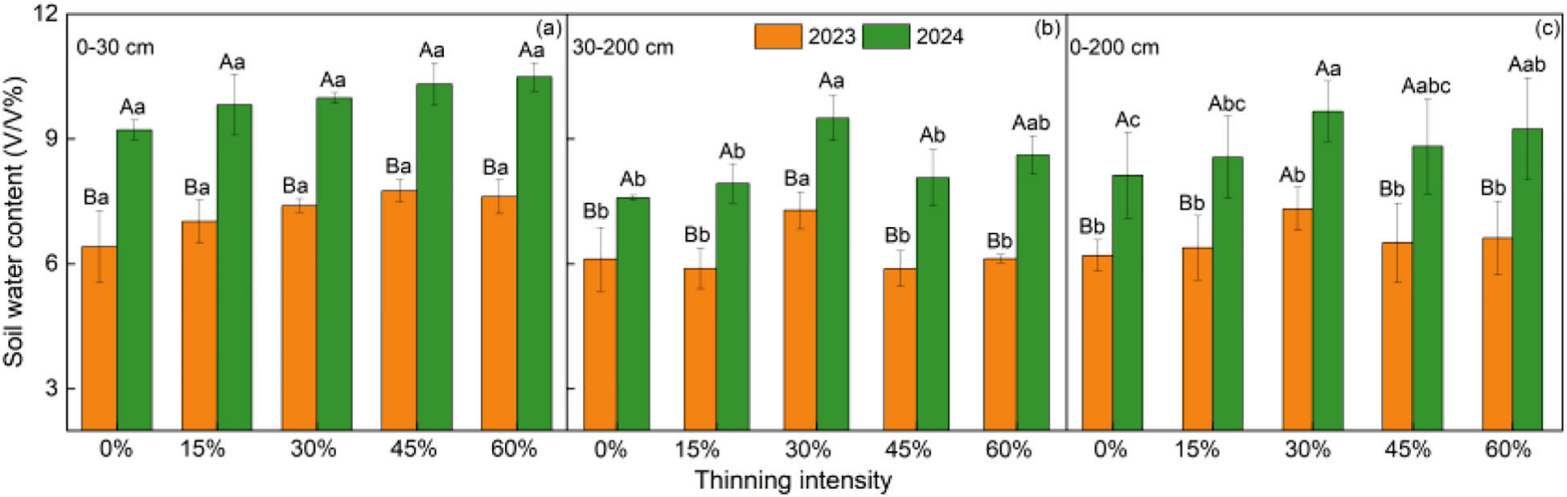
Mean SWC across thinning intensities and soil depths during the monitored observation period in 2023 and 2024. Note: Different uppercase letters denote significant differences between years within a given thinning intensity, and different lowercase letters denote significant differences among thinning intensities within the same year (*P* < 0.05).

The effect of thinning on SWC was most pronounced in the deep layer, where the 30% thinning treatment consistently maintained the highest water content in both study years (**Figure 3** and **4**). Overall, all thinning treatments increased SWC relative to the 0% thinning control, with mean profile SWC increasing by 4.96–18.06% in the dry year (2023) and by 5.33–18.87% in the normal year (2024) (**Figure 3**). Although SWC was 31.1–37.8% higher in 2024 than in 2023 across all treatments, treatment-related differences were more pronounced in the 30–200 cm layer than in the 0–30 cm layer (**Figure 4**). These results suggest that thinning-related differences in SWC were mainly expressed in deep soil water storage rather than surface SWC, highlighting the key role of the deep layer in distinguishing treatment effects.

### 3.2 Temporal dynamics of SWC under different thinning intensities

**Figure 5:**
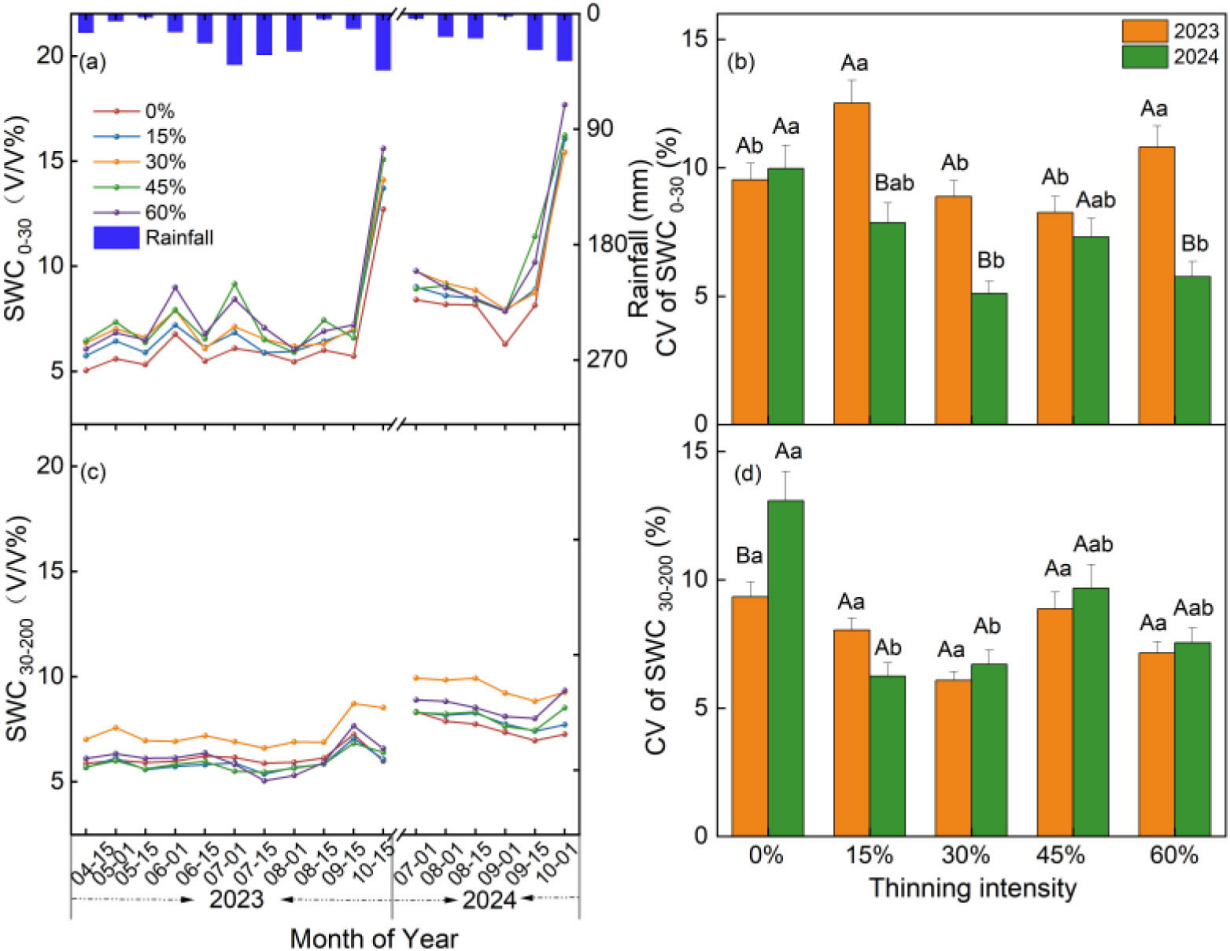
Temporal changes in rainfall, soil water content (SWC), and coefficient of variation (CV) from 2023 to 2024. (a) Temporal changes in rainfall and surface SWC (0–30 cm). (b) Coefficient of variation of SWC in the 0–30 cm layer. (c) Temporal changes in deep SWC (30–200 cm). (d) Coefficient of variation of SWC in the 30–200 cm layer. Different uppercase letters denote significant differences between years within the same thinning intensity, and different lowercase letters denote significant differences among thinning intensities within the same year (P < 0.05).

Temporal SWC responses differed markedly between soil layers, and the 15% and 30% thinning treatments generally showed lower variability in deep SWC in both study years (**Figure 5**). Monthly SWC in the 0–30 cm layer fluctuated synchronously with seasonal rainfall and rose sharply in early October in both years, whereas SWC in the 30–200 cm layer exhibited much lower temporal variability (**Figures 5A, C**). Compared with 2023, surface SWC in 2024 showed lower temporal variability under all thinning treatments except the control. The 15% and 30% thinning treatments exhibited the lowest CVs in the deep layer during the monitored observation period (*P* < 0.05; **Figures 5B, D**). These patterns indicate that the temporal effect of thinning was expressed chiefly as reduced variability in deep soil water storage rather than as a change in rainfall-synchronous surface fluctuations.

### 3.3 Relating the effects of thinning on SWC to environmental variables

**Figure 6:**
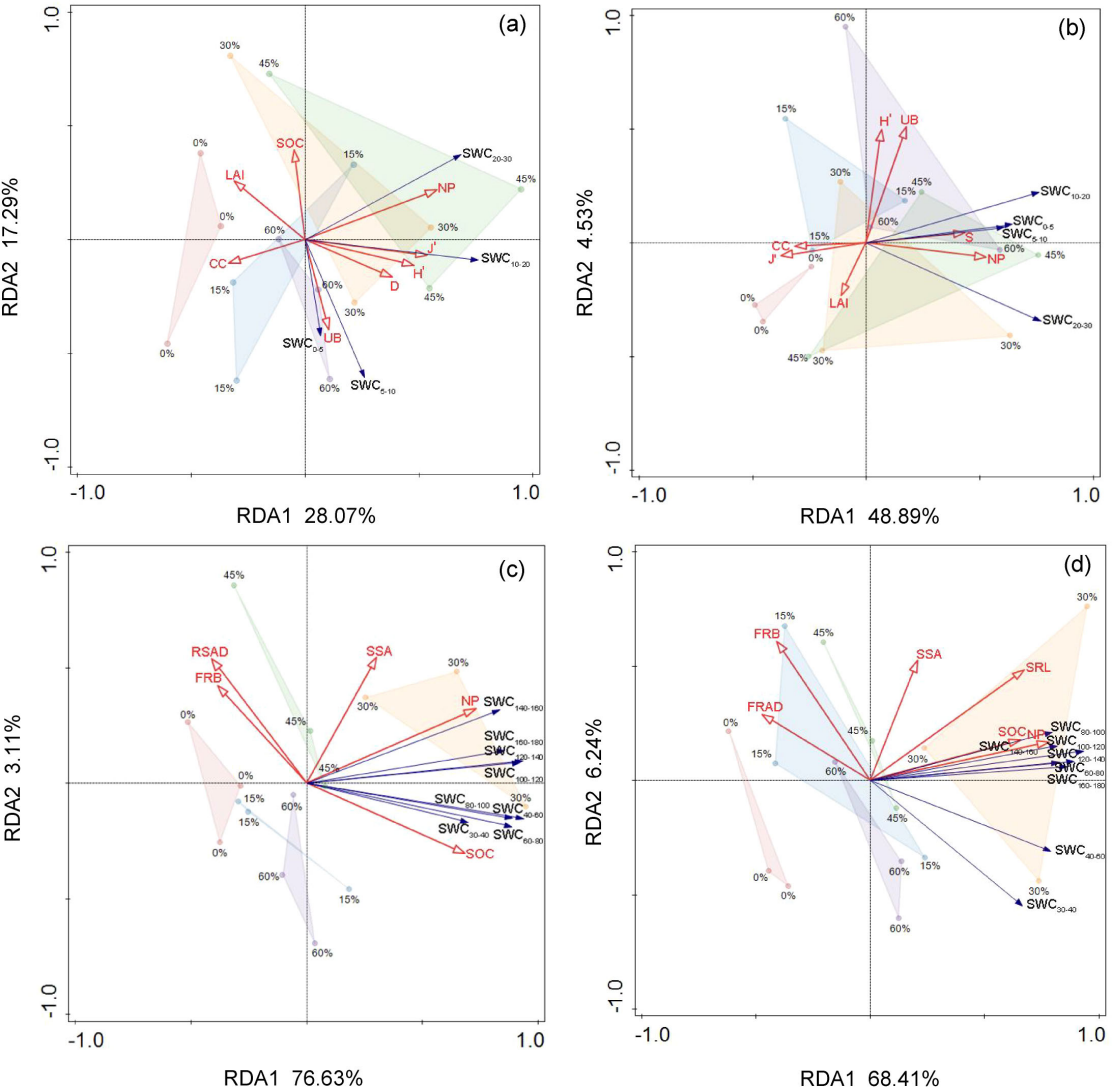
Redundancy analysis (RDA) ordination biplots showing the relationships between SWC in surface (0–30 cm; **(A, B)**) and deep (30–200 cm; **(C, D)**) soil layers with explanatory variables in 2023 **(A, C)** and in 2024 **(B, D)**. Different color patches represent different thinning intensities. Detailed quantitative results for the first two RDA axes, including eigenvalues, percentages of variance explained, and correlations with explanatory variables, are provided in **Supplementary Tables 1–S4**. LAI, leaf area index; SOC, soil organic carbon; NP, net precipitation; *S*, Patrick richness index; *H′*, Shannon–Wiener diversity index; *D*, Simpson index; *J*, Pielou index; UB, understory biomass; CC, canopy closure; SSA, specific surface area; SRL, specific root length; FRB, fine root biomass; FRAD, fine root average diameter; RSAD, root surface area density.

RDA showed that relationships between SWC and environmental variables varied by soil depth, with a larger proportion of SWC variation explained in the deep layer than in the surface layer in both study years (**Figure 6**). The first two axes explained 45.36% and 53.42% of the variation in surface layer SWC in 2023 and 2024, respectively, compared with 79.64% and 74.66% for deep SWC (**Supplementary Table 1** and **S2**; **Figure 6**). In the surface layer, SWC was positively correlated with net precipitation in both years, being positively associated with understory diversity in 2023 and with species richness in 2024, while canopy closure remained negatively correlated with SWC in both years (**Supplementary Table 3**; **Figures 6A, B**). In contrast, in both years, deep SWC exhibited a strong positive correlation with net precipitation and soil organic carbon (**Figures 6C, D**). In addition, deep SWC in 2024 was also positively associated with finer root traits, including specific root length and specific surface area (**Supplementary Table 4**; **Figure 6D**). Collectively, these results indicate that the relationships between SWC and environmental variables varied more between years in the surface layer, whereas deep SWC remained primarily associated with net precipitation and soil organic carbon in both study years.

## 4 Discussion

### 4.1 Profile-scale and layer-specific SWC responses to thinning

In both study years, profile SWC peaked under the 30% thinning treatment, and treatment-related differences were expressed mainly in the deep layer rather than at the surface. This result suggests that the effect of thinning on soil water storage was governed by a balance between reduced stand-level water consumption and compensatory water use by residual trees. By reducing tree density, thinning lowers total stand transpiration and can thereby increase soil water storage (Simonin et al., 2007). However, as thinning intensity increases, residual trees may undergo crown expansion and growth release, which increases water use per tree and progressively offsets the stand-level water saving (Bréda et al., 1995; del Campo et al., 2022). As a consequence, SWC did not increase continuously with thinning intensity, but instead peaked under moderate thinning. Contrary to our initial hypothesis, the response of profile SWC to thinning was similar in the dry and normal years. This similar response further indicates that interannual precipitation mainly altered the baseline level of soil water availability, whereas thinning regulated the relative gain in SWC through its influence on stand water use (Rahmati et al., 2024). Because treatment-related differences were concentrated in the deep layer, where water content is less sensitive to short-term rainfall fluctuations, the thinning intensity that maximized profile SWC remained unchanged in both study years. Overall, these findings indicate that moderate thinning was sufficient to reduce stand water demand without inducing the stronger compensatory water use that likely occurred under heavier thinning.

RDA indicated that the relationships between SWC and environmental variables differed by soil depth (**Figure 6**; **Supplementary Table 1** and **S4**). Surface SWC was associated primarily with near-surface water inputs and understory vegetation, whereas deep SWC was associated primarily with variables related to profile water storage. In the surface layer, SWC was positively associated with net precipitation in both years, which is consistent with the rapid response of surface soil to rainfall inputs after thinning reduced canopy interception and increased throughfall (Ma et al., 2020; Fischer-Bedtke et al., 2023). Surface SWC was also positively associated with understory diversity, likely because canopy opening after thinning increases understory light availability and space, which can increase understory cover and diversity. Higher understory diversity is often accompanied by greater ground cover and litter continuity, which reduces post-rainfall evaporative losses and helps retain water in the 0–30 cm layer (Livne-Luzon et al., 2025). In contrast, deep SWC was positively associated with net precipitation and soil organic carbon in both years, indicating that water storage in the 30–200 cm layer depended more strongly on downward infiltration and soil water-holding capacity. Thinning can enhance deep water storage by reducing stand-level transpiration demand, while higher soil organic carbon can improve aggregation and pore structure, thereby increasing the proportion of infiltrated water retained at depth (Molina and del Campo, 2012; Wang et al., 2021). Notably, in the normal year (2024), deep SWC also covaried positively with fine root specific root length. A higher fine root specific surface area is commonly accompanied by more extensively distributed fine roots and a larger contact area between roots and the surrounding soil. This can promote the formation of more root channels and preferential flow pathways under higher precipitation, facilitating deeper infiltration and increasing the amount of water stored in the deep layer (Qi et al., 2020). Overall, these depth-dependent relationships suggest that thinning influenced surface SWC mainly through rainfall redistribution and understory cover, whereas its effects on deep SWC were mediated more by profile-scale water storage processes.

### 4.2 Temporal stability of surface and deep SWC under thinning in the two study years

Surface SWC was consistently more variable than deep SWC across thinning intensities and in both study years, reflecting the greater sensitivity of the 0–30 cm layer to rainfall pulses and subsequent evapotranspirational losses. In the dry year, surface SWC variability increased after thinning, particularly under the 15% and 60% treatments, suggesting that more heterogeneous throughfall and infiltration under light thinning and stronger evaporative loss under heavy thinning amplified short-term fluctuations in the surface layer (André-Alphonse et al., 2023; Chen and Sivapalan, 2020; Doukalianou et al., 2022). In the normal year, by contrast, surface SWC variability declined under thinning and was lowest under the 30% treatment, indicating that moderate thinning provided a more favorable balance between increased water input to the forest floor and reduced surface water loss (Rascón-Ramos et al., 2021; Sun et al., 2016). Notably, surface SWC increased sharply at the end of the growing season in both years (**Figure 5A**), reflecting non-growing season rainfall recharge, reduced water use and evaporation under cooler conditions, and litter-induced evaporation suppression (Balugani et al., 2017; Park et al., 1998). However, the persistence of this recharge and its potential to alleviate subsequent drought remain unclear and require long-term monitoring.

Thinning reduced the temporal variability of deep SWC in both years, and this reduction was more pronounced in the normal year than in the dry year. Compared with the surface layer, deep SWC changed more gradually because water stored at depth was buffered against short-term rainfall events and evaporative losses, resulting in a greater buffering capacity in the deep layer. In the dry year, the reduced variability of deep SWC after thinning likely reflected lower stand-level extraction of deep SWC, which alleviated episodic drawdown during drought and thereby dampened temporal fluctuations (Jia et al., 2017). In the normal year, more favorable antecedent water conditions and more frequent rainfall likely enhanced the continuity of deep recharge, reducing the development of prolonged deep water deficits and further stabilizing deep SWC (Tashie et al., 2016; Lai et al., 2016; Zignol et al., 2025). Deep SWC under 15% and 30% thinning remained comparably stable across both years, suggesting that light-to-moderate thinning maintained a relatively balanced deep SWC regime. This result further indicates that, under light-to-moderate thinning, reductions in deep SWC consumption were sufficient to limit episodic depletion without disrupting the continuity of deep recharge. Overall, deep SWC changed more slowly than surface SWC, with variability differing by thinning intensity and hydrological year, providing a hydrological basis for optimizing thinning intensity on Loess Plateau plantations.

### 4.3 Implications for reasonable thinning intensity determination

In the semi-arid regions, limited precipitation renders SWC the primary limiting factor for plantation growth and vegetation recovery (Huang and Shao, 2019). Consequently, the soil water carrying capacity for vegetation is widely used by researchers to quantitatively evaluate sustainable stand density and mitigate the risk of deep soil desiccation. Previous studies suggest that the suitable density for Chinese pine plantations in this region is generally about 1100–1600 stems ha^-1^ (Zhang et al., 2023). However, our findings revealed that thinning intensities exceeding 30% failed to effectively improve profile SWC. In contrast, retaining approximately 2000 stems ha^-1^ (30% thinning) significantly increased deep SWC by 19.14–25.26% and was associated with lower temporal variability in the two study years. Notably, even though this residual density exerts a positive effect on SWC, it still exceeds the soil water carrying capacity for vegetation in this region. This implies that multiple thinning operations are required to optimize the stand structure of these plantations, with the initial thinning intensity maintained at approximately 30%.

At the operational level, the core objective is to translate these phased targets into actionable silvicultural practices. For the initial thinning, a moderate intensity of 30% should be adopted as the primary approach, with a residual stand density of approximately 2000 stems ha^-1^ maintained to enhance soil water availability and stabilize SWC dynamics. Notably, thinning interventions should follow an adaptive management strategy based on hydrological year types. In normal years, stand structure optimization serves as the core objective, where thinning and understory tending measures are implemented to efficiently improve soil water storage by leveraging hydrological windows with abundant precipitation. In contrast, in dry years, the focus shifts to soil water conservation, where large-scale thinning operations are suspended to reduce the ineffective water consumption of trees during the non-growing season. During each thinning intervention, managers should prioritize the selective removal of suppressed, small-diameter, diseased, and senescent trees, while retaining well-formed dominant individuals and preserving canopy continuity as well as litter cover on the forest floor. Post-thinning assessments should emphasize continuous profile-scale soil water monitoring to characterize the spatiotemporal dynamics of SWC with depth and over time, as well as to quantify its temporal stability. A second thinning can be implemented if two specific conditions are met: either profile SWC remains persistently below field capacity in a normal year, or obvious water-stress symptoms coincide with a significant slowdown in diameter growth. This adaptive, stepwise approach to subsequent thinning leverages post-thinning ecosystem feedbacks, allowing for dynamic refinement of management strategies rather than rigid adherence to predefined stand density targets. Notably, this study focused primarily on SWC dynamics during the growing season. However, because SWC observations from May to June 2024 were unavailable, the present dataset may not have fully reflected early-growing-season fluctuations in SWC, particularly in the surface layer. This may have affected the estimation of seasonal mean SWC and CV in 2024, as well as their comparability with the complete 2023 dataset. These limitations indicate that a more comprehensive evaluation of the effects of thinning will require both more complete seasonal monitoring and a broader set of ecological indicators. Accordingly, to advance multidimensional ecological restoration in plantations, future studies should integrate comprehensive functional indicators, such as understory vegetation and carbon cycling, to optimize thinning regimes in terms of both intensity and frequency. This approach will ultimately enhance sustainable plantation management across the Loess Plateau and similar water-limited regions.

## 5 Conclusions

This study quantitatively assessed depth-resolved SWC responses to thinning in two study years representing a dry year (2023) and a normal year (2024) in Chinese pine plantations on the Loess Plateau. Overall, our results show that thinning increased SWC. Thinning increased mean profile SWC by 4.96–18.06% in the dry year (2023) and by 5.33–18.87% in the normal year (2024). Although SWC was higher in 2024 than in 2023, the response of SWC to thinning was broadly similar in the two study years, and 30% thinning produced the strongest increase in deep SWC (30–200 cm; +19.14–25.26%). In 2024, thinning significantly enhanced the temporal stability of deep SWC, and the 15% and 30% thinning treatments showed relatively low CV values during the monitored observation period. RDA indicated that SWC was positively associated with net precipitation in both soil layers and years, with additional positive associations with diversity, soil organic carbon, and fine-root traits. Although the post-thinning density still exceeds the local soil water carrying capacity for vegetation range, over 30% thinning (residual density ≈ 2000 stems ha^-1^) did not improve SWC in the dry year (2023) and increased SWC instability. Therefore, we propose setting the first thinning threshold at about 30% (retaining ≈ 2000 stems ha^-1^), followed by stepwise reductions to gradually approach the recommended range based on the soil water carrying capacity for vegetation. These findings provide a practical basis for setting thinning thresholds to improve SWC sustainability in semi-arid plantations.

## Data Availability

The raw data supporting the conclusions of this article will be made available by the authors, without undue reservation.
