# Supplementary material for: Moderate thinning enhances soil water and its temporal stability in Chinese pine plantations on the semi-arid Loess Plateau of China
Source: Front Plant Sci. 2026 Apr 29;17:1805676. doi: 10.3389/fpls.2026.1805676 (PMC13168105; doi:10.3389/fpls.2026.1805676)
Supplement: Supplementary file 1 [file Table1.docx]

Table S1. Eigenvalues and percentages of variance explained by the first two RDA axes in the surface layer (0–30 cm) for the dry year (2023) and normal year (2024).

| Surface layer | Dry year (2023) | |  | Normal year (2024) | | |
| --- | --- | --- | --- | --- | --- | --- |
|  | Axis | |  | Axis | | |
|  | 1 | 2 |  | 1 | 2 |  |
| Eigenvalue | 0.2807 | 0.1729 |  | 0.4889 | 0.0453 |  |
| Explained variance (%) | 28.07 | 17.29 |  | 48.89 | 4.53 |  |
| Cumulative variance (%) | 28.07 | 45.36 |  | 48.89 | 53.42 |  |
| Explained fitted variation (%) | 56.13 | 34.58 |  | 87.49 | 8.11 |  |

Table S2. Eigenvalues and percentages of variance explained by the first two RDA axes in the deep layer (30–200 cm) for the dry year (2023) and normal year (2024).

| Deep layer | Dry year (2023) | |  | Normal year (2024) | | |
| --- | --- | --- | --- | --- | --- | --- |
|  | Axis | |  | Axis | | |
|  | 1 | 2 |  | 1 | 2 |  |
| Eigenvalue | 0.7653 | 0.0311 |  | 0.6841 | 0.0624 |  |
| Explained variance (%) | 76.53 | 3.11 |  | 68.41 | 6.25 |  |
| Cumulative variance (%) | 76.53 | 79.64 |  | 68.41 | 74.66 |  |
| Explained fitted variation (%) | 92.41 | 3.75 |  | 86.31 | 7.88 |  |

Table S3. Correlation coefficients between RDA ordination axes and explanatory variables in the surface layer (0–30 cm) for the dry year (2023) and normal year (2024)

| Surface layer | Dry year (2023) | |  | Normal year (2024) | |
| --- | --- | --- | --- | --- | --- |
|  | Ordination axis | |  | Ordination axis | |
|  | 1 | 2 |  | 1 | 2 |
| NP | 0.422 | 0.159 |  | 0.431 | 0.385 |
| S | 0.390 | -0.051 |  | 0.355 | 0.026 |
| Hʹ | 0.348 | -0.083 |  | 0.055 | 0.296 |
| D | 0.279 | -0.121 |  | — | — |
| J | 0.390 | -0.050 |  | -0.305 | -0.034 |
| UB | 0.076 | -0.290 |  | 0.145 | 0.303 |
| CC | -0.244 | -0.076 |  | -0.255 | -0.009 |
| LAI | -0.226 | 0.188 |  | -0.091 | -0.137 |
| SOC | -0.034 | 0.287 |  | — | — |

Table S4. Correlation coefficients between RDA ordination axes and explanatory variables in the deep layer (30–200 cm) for the dry year (2023) and normal year (2024)

| Deep layer | Dry year (2023) | |  | Normal year (2024) | |
| --- | --- | --- | --- | --- | --- |
|  | Ordination axis | |  | Ordination axis | |
|  | 1 | 2 |  | 1 | 2 |
| NP | 0.709^**^ | 0.232 |  | 0.727^**^ | 0.147 |
| SOC | 0.663^**^ | -0.221 |  | 0.612^*^ | 0.166 |
| SSA | 0.295 | 0.395 |  | 0.194 | 0.420^*^ |
| SRL | — | — |  | 0.628^*^ | 0.453 |
| FRB | -0.372 | 0.306 |  | -0.377 | 0.565^*^ |
| FRAD | — | — |  | 0.194 | 0.420^*^ |
| RSAD | -0.396 | 0.389 |  | — | — |

*, the correlation with the redundancy analysis (RDA) ordination axis is significant at the 0.05 level. **, the correlation with the RDA ordination axis is significant at the 0.01 level. Values are correlation coefficients between retained explanatory variables and the first two RDA axes. Variables measured but excluded due to multicollinearity (high VIF) are indicated by ‘—’. Notes: LAI, leaf area index; SOC, soil organic carbon; NP, net precipitation; *S*, Patrick index; *Hʹ*, Shannon-wiener index; *D*, Simpson index; *J*, Pielou index; UB, understory biomass; CC, Canopy Closure; SSA, specific surface area; SRL, specific root length; FRB, fine root biomass; FRAD, fine root averaged diameter; RSAD, root surface area density.
